# Spaceflight and Medical Microbiology: Possible Implications for Standard Infection Diagnostics and Therapy

**DOI:** 10.3390/life15111757

**Published:** 2025-11-15

**Authors:** Alessa Lalinka Boschert, Stefan Leuko, Carolin Luisa Krämer, Katharina Siems, Yen-Tran Ly-Sauerbrey, Franca Arndt

**Affiliations:** 1 MVZ Laboratory Dr. Limbach & Colleagues eGbR, 69126 Heidelberg, Germany; 2 Applied Aerospace Biology, Institute of Aerospace Medicine, German Aerospace Center, Linder Hoehe, 51147 Cologne, Germany; 3 Applied Sciences, University of Applied Sciences Bonn-Rhein-Sieg, Von-Liebig-Straße 20, 53359 Rheinbach, Germany

**Keywords:** microbiological diagnostics, manned missions, infections, long-term missions

## Abstract

Infections pose a major risk during long-term human spaceflight missions. By applying standard procedures in medical microbiology to a hypothetical urinary tract infection during a Mars-bound flight, important practical aspects become apparent. From infection diagnostics to antimicrobial treatment during spaceflight, issues include technological constraints, the lack of breakpoints, and epidemiological data. A potential solution is a combination of data acquisition, artificial intelligence, individualized medicine, novel diagnostic tools, and antimicrobial strategies. This work takes an exploratory approach to highlight challenges and potential directions in developing diagnostic strategies for long-term space missions.

## 1. Introduction

Microbiology in human spaceflight is, and has been in the past, an important field of research, with one of its goals being to maintain human health [1,2,3]. Initially, most studies concentrated on one of the following four specialty areas: physiological changes, pathogen behavior, antimicrobial substances, or the spaceflight environment. However, the interconnectivity of these four areas has been studied in more detail in recent years [4,5,6]. Also, infection prevention has come into sharp focus [7]—an important development that is pivotal for spaceflight.

Extensive pre-flight protocols, including but not limited to pre-mission health stabilization, vaccination, and preventive dental care [8], as well as weekly cleaning protocols and microbial monitoring aboard the International Space Station (ISS) [9], aim to prevent the occurrence of infections in the first place. This ensures that the risk of infection is minimized. Yet, due to human and environmental microbiota [10,11], in combination with the extreme space environment, the risk cannot be fully eliminated. Reactivation of latent herpes virus infections [12,13], as well as other infections, such as skin infections [14], are well documented. It is particularly notable that most of these incidents occur during the first month of the mission [14]. This implies that these incidents are likely to arise during travel to Mars or while being on the planet itself. Therefore, infection management during long-term space missions is already part of extensive considerations [15].

In the following, we would like to engage in a thought experiment to point out some of the potentially pragmatic issues that occur when considering infections in spaceflight. It is important to bear in mind that, hence, some conclusions will be of a speculative nature. However, it will hopefully give a new perspective on the current challenges. An infection and its step-by-step diagnostics will be approached as it may happen in a “normal” medical setting. As described above, various infections can occur under spaceflight conditions. Amongst others, a total of 23 medical events regarding the genitourinary system were reported during space shuttle missions from 1981 to 1998 [16]. One prominent infection that happened during spaceflight was a device-associated urinary tract infection (UTI) caused by *Pseudomonas* spp. in astronaut Fred Haise during the Apollo 13 mission [17]. Generally, there seems to be evidence that the growth of aerobic bacteria, such as *P. aeruginosa*, may be favored under spaceflight conditions [18,19]. Moreover, *P. aeruginosa* was detectable in water sources aboard the ISS [20]. Furthermore, sepsis, primarily urosepsis, has been identified as an important medical emergency with high potential to cause evacuation aboard the ISS [21]. In addition, the close interaction between the bladder, the gut microbiota, and their role in infection [22], in combination with the well-established immunosuppression caused by the space environment [23], makes an endogenous infection of the urinary tract a potential risk during long-term missions. Therefore, in our thought experiment, we will use a UTI caused by *P. aeruginosa* as an example of an infection that could occur during a flight to Mars.

**Figure 1:**
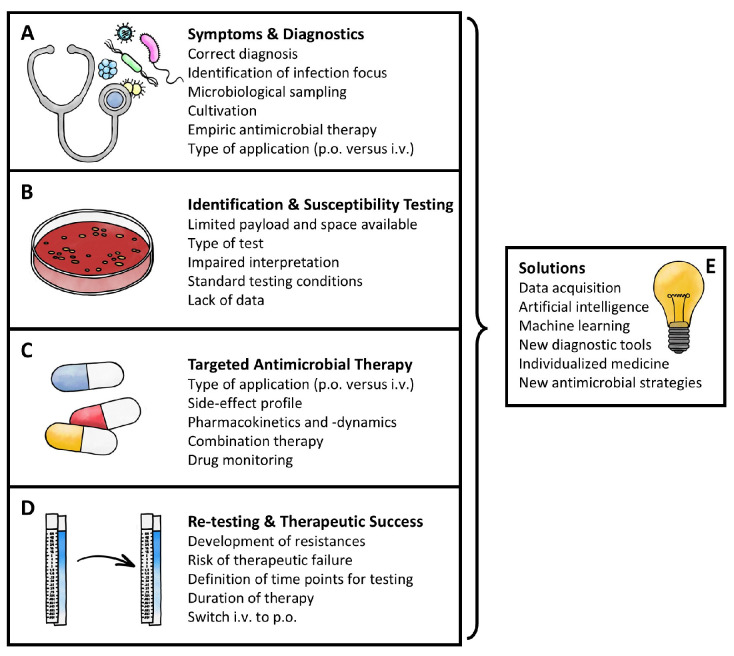
Summary of the most important issues during infection diagnostics and treatment. Boxes (**A**–**D**) refer to the major steps needed, while box (**E**) points out potential solutions [p.o.: per os—oral application; i.v.: intravenous] (Created with Adobe Fresco).

The following sections of this essay outline the different stages of our hypothetical UTI, from the initial symptoms and diagnosis to identifying the pathogen and testing its susceptibility to antibiotics, and finally administering targeted antimicrobial therapy and re-testing it to evaluate therapeutic success. For each stage, we highlight the specific issues that arise (illustrated in Figure 1) and evaluate possible strategies to overcome these challenges.

## 2. Symptoms and Diagnostics

In the assumed scenario, a team member presents with increasing malaise, elevated temperature, chills, and a burning sensation when urinating during the second week of the mission. For the sake of simplicity, the sum of the symptoms chosen points quite unequivocally towards a UTI. While the last symptom indicates the infection’s focus in the exemplary scenario here, it might not necessarily be present in other cases. Unspecific symptoms often, especially, require not only a thorough examination but also a sampling of various microbiological materials to identify the origin of infection.

While medical training is an integral part of astronaut training, the initial challenge remains of diagnosing an infection and identifying its focus. In addition, a local infection needs to be differentiated from progress towards a systemic infection. Even on Earth, this is not always as straightforward as it seems. A perfect example to illustrate this is the diagnosis of sepsis. Sepsis is defined as a “life-threatening organ dysfunction caused by a dysregulated host response to infection” [24]. However, various biochemical, microbiological, and clinical features need to be considered. The non-specific symptoms often render the diagnosis difficult [25,26], especially in the elderly [27]. The latter aspect also bears some consideration, since the assessment of the elderly does not only rely on the chronological, but also the biological age [28]. Therefore, it is important to understand how the harsh space environment affects biological aging [29], in order to elucidate what symptoms to expect.

Like in some clinical presentations, infection diagnostics may be further impeded during space missions: Due to immunosuppression in astronauts [23], potential symptoms might present as less specific or less severe, harboring the risk of misinterpretation and a fast progression to critical, systemic infections. One potential solution for sepsis and infection diagnostics that is also suited for the space environment is point-of-care testing (POCT) [30]. These tests, relying on biomarkers, not only allow for a fast turn-around time but are usually easy to handle, thereby reducing the potential contamination risk within the spacecraft. POCT has been proven to support diagnosis, especially in infections difficult to identify. For example, procalcitonin allows for easier differentiation between infected and non-infected diabetic foot ulcers when the clinical signs are ambiguous [31]. Given the above-discussed potential regarding atypical clinical signs of infection during space missions, POCT might substantially aid in early diagnosis and, thus, prevent progression from a local to a systemic infection.

As published in 2024 by Rea et al., a flow cytometer has been successfully applied aboard the ISS [32]. This is of particular interest when looking at sepsis biomarkers: CD64 is a biomarker that has been shown to reliably distinguish sepsis from systemic inflammatory response syndrome. In the studies published so far, it has been assessed by flow cytometry [33], hence making it a perfect candidate for POCT in space missions.

In our scenario, a UTI is suspected, and now there are several initial problems that need to be addressed (Figure 1A):

First, sampling needs to happen, and the causative agent needs to be identified. Even with the current progress in medical microbiology and the development of new methods, such as microfluidics and genotypic resistance testing [34], currently, cultivation is still the gold standard to proceed. Yet, even on Earth, sampling can often prove difficult [35] with the risk of contamination by the mucosal flora and, hence, difficult interpretation. In space, where fluids present a potential hazard, this might be even more aggravated, not least in obtaining a sterile sample. After having succeeded, the sample needs to be cultivated, usually on media comprising universal, selective, and chromogenic agar [36]. Growth is then assessed after 24 and 48 h, respectively. Some studies have described an increased proliferation rate of bacteria [5] under spaceflight conditions. Apart from the possible implications for the infection itself, this might generally allow for easy cultivation. However, it might necessitate shorter incubation periods, as high growth densities might impede the differentiation between the potential pathogens from the commensals of the mucous membrane. In addition, in some cases, the latter task may be easily achieved, but might be further complicated by the increased growth rates in others, especially when thinking of a low bacterial count UTI [37,38]. For the interpretation of the cultivation results, medical expertise might be required. During short-term or Earth-orbit missions, it would be easy to receive direct support via radio interface. However, depending on the exact location when traveling to Mars, radio communication is delayed [39]. One potential solution lies in automation: More and more systems have been developed that not only automatically cultivate the samples, but are, to a certain degree, also able to interpret them [40].

In general, important questions regarding the cultivation media remain: Which media should be taken on a long-term mission, how will they change due to their exposure to the spaceflight environment, and how will it affect their shelf life? This also interlinks with another important microbiological sample for infection diagnostics: Blood cultures. Bacterial count during bloodstream infections is low, being around 1–10 colony-forming units per mL of blood [41]. Therefore, enrichment is required before being able to evaluate growth. This usually comprises a certain amount of automation, e.g., via the widely used BACTEC^TM^ [42]. Yet, all of these systems used in routine laboratory diagnostics are not suited for spaceflight, not least due to their size and unknown functionality under microgravity. However, technologies for fast molecular diagnostics are evolving rapidly [43,44], offering possible solutions to this issue. Similarly, POCT may also contribute to UTI diagnostics [45].

Proceeding in the thought experiment, as already stated, there is always the risk of fast progression from a local to a systemic infection. To prevent this, an empiric antibiotic therapy should be started. This relies on choosing an antimicrobial substance that will most likely cover the most common pathogens, including their current resistance behavior, for the respective infection. In other words, for empiric treatment of any given infection, the local and current epidemiological situation, differing between or even within hospitals, needs to be accounted for.

This is an immense challenge when applying this principle to crewed spaceflight. First of all, epidemiological starting points vary substantially between crews comprising different members, for example, due to differing home countries [46]. In addition, there remains a general lack of data. Many studies focus on the molecular mechanisms of resistance in space or simulation models, mainly concentrating on model organisms [47,48,49,50]. Understanding the basic mechanisms of the microbial response to spaceflight is of pivotal importance, for example, in the assessment of repeated susceptibility testing and therapy duration. However, in order to develop a Gaussian distribution, information on a large number of strains within one species and their altered resistance behavior is required. Only then can a decision be made on potential empirical therapies, and moreover, this is also important for antimicrobial susceptibility testing. This is a topic that will be discussed later.

Additionally, due to the relatively low number of astronauts, the number of reported infections in space is limited [14,18,19]. This makes it difficult to assess potential differences in the causative agents. Yet, several assumptions can be made in this regard: Many infections are endogenous, i.e., derive from opportunistic pathogens of the physiological microbiota of the astronauts. Any changes in the microbiome, such as the already mentioned potential selection for aerobic bacteria [18,19], will affect the expected pathogen spectrum. These effects can be direct due to colonization, but also indirect because of immunomodulation [51,52]. In addition, the confinement of the spacecraft with only a limited number of inhabitants in combination with the selective pressure of the space environment might point towards a shift in the microbial spectrum towards that of nosocomial pathogens, including *P. aeruginosa*. After all, the described setting is similar to that of an intensive care unit, such as access for only a limited number of people and immunocompromised patients. Some additional risk factors—in the case of the Apollo missions, the urinary collection device—further support this argument.

Taking these aspects into account in our hypothetical UTI, the situation is as follows: If the most prevalent pathogen of community-acquired UTIs, *Escherichia coli*, is expected, and the antibiotics suggested by the respective guidelines [53,54], such as pivmecillinam, should be effective, while still bearing the local resistance situation in mind. However, assuming a shift in the microbial community, e.g., increased abundance of *P. aeruginosa*, intrinsic resistance to most of the commonly used antibiotics is possible. As the issue is not known for certain at this point, but changes are to be expected, the easiest option would be to decide on a broad-spectrum antimicrobial substance. This would ensure coverage of most pathogens. However, the high efficacy of these antibiotics comes with the trade-off of resistance development [55]. Due to the already confined space, an inter- and intra-species transfer of antimicrobial resistance (AMR) genes is highly likely, with these genes already being found aboard the ISS [56]. Hence, initial empiric antimicrobial therapy should cover a microbial spectrum as narrow as possible to reduce the risk of developing resistance.

These considerations are further complicated by the potential microbial response to the spaceflight environment, which is not uniform: While some species, such as *Salmonella typhimurium*, exhibit increased virulence [57], that of others, such as *Enterococcus faecalis* [48], is reduced. This aspect also needs to be considered for empiric therapy.

Finally, a decision must be made on the type of application, oral (p.o.—per os) or intravenous (i.v.). One important feature to bear in mind for this decision is the actual nature of the infection: Is it a lower urinary tract infection or has it already developed towards a urosepsis? Other factors important for reaching a decision on this matter will be discussed in the section on targeted therapy.

## 3. Identification and Susceptibility Testing

Once the pathogen has been successfully cultivated, the next steps are the identification and susceptibility testing of the organism. When boiling down potential queries of this phase, some, as described below, are similar to the issues above, while others are, quite simply, the payload and space availability [58] (Figure 1B).

There are some biochemical tests, such as in the case of *P. aeruginosa* testing positive for oxidase, that may point towards the correct bacterial species. Yet, the current standard of identification is performed by Matrix-assisted laser desorption/ionization time-of-flight (MALDI-TOF) [59], i.e., mass spectroscopy. While this is a reliable and quick identification method, current devices require a huge amount of space, an important resource that is limited in spacecrafts. However, there are several new technologies that would allow for smaller devices [60,61]. Using the current methods, preparation of the colonies would also pose a potential fluid hazard, but it could be performed, for example, in a glove box, as the one already present on the ISS [62].

The next two problems that we would like to point out both focus on the susceptibility testing itself, considering altered diffusion under microgravity, and hence a deviating interpretation of minimal inhibitory concentration (MIC) values for susceptibility. For susceptibility testing, there are several possible methods in medical microbiology: Automated systems, such as Vitek 2 [63] or Phoenix [64], agar diffusion tests, and microdilution. Choosing the method depends, amongst others, on the species. The automated systems, for example, are not feasible for obligate anaerobic bacteria or may be difficult to deploy in species that have a great variability in their growth, such as *P. aeruginosa* [65]. This is because these systems largely depend on measuring the optical density. Arbitrary growth changes, such as mucous production and slow growth in *P. aeruginosa*, can make results unreliable. Disk diffusion methods only allow for indirect extrapolation of the resistance via a measured inhibition zone [66]. Microdilution methods are labor-intensive, but they remain a state-of-the-art testing method in clinical diagnostics for most species. However, all these systems have one characteristic that presents a hindrance in space: In one way or another, they rely on diffusion, whether it be within a fluid or within a solid, which is altered in spaceflight due to microgravity conditions [67,68].

Hence, the testing procedure itself as well as the interpretation might be impaired. Any results may differ between spaceflight and Earth, i.e., different MICs might indicate the same susceptibility level.

This directly leads to the next problem: The ubiquitous lack of data. Measuring the MIC is insufficient on its own. It also requires interpretation to determine whether a certain concentration indicates susceptibility or resistance to a certain antibiotic. This is usually performed by institutions, such as the European Committee of Antimicrobial Susceptibility Testing (EUCAST) or the Clinical and Laboratory Standards Institute (CLSI). In order to achieve this, large amounts of clinical, pharmacological, and epidemiological data need to be acquired [69]. Comparing the measured MICs to clinical data, including but not limited to the target species, infection type, pharmacodynamics, and kinetics, allows for the definition of clinical breakpoints. MICs measured below this breakpoint indicate that a therapeutic success is highly likely. However, being able to draw these conclusions relies on given standard testing conditions that need to be adhered to, such as a defined temperature range, humidity, incubation time, and the bacterial suspension itself [70]. As these methods are developed on Earth, they are inevitably standardized for a 1 g environment. Hence, transferability of the breakpoints may be limited between the microgravity environment, or partial gravity environments such as on the Moon (0.16 g) or Mars (~0.38 g), and in-flight testing [71]. The gravitational conditions of Mars have already been simulated by centrifuge experiments aboard the ISS [72]. Susceptibility testing in centrifuges may provide initial indications of MIC interpretations.

The issue can be exemplified by previous experiments in space: Increased MIC values were measured when testing *Staphylococcus aureus* and *E. coli* during spaceflight [5,73]. This could be interpreted as an increased antibiotic resistance; however, the testing conditions, as well as the previously described changes in growth, need to be considered. The observed MICs are most likely influenced by the changed diffusion, altered shear forces, and changed growth, such as the aforementioned potential increase in bacterial proliferation [5], caused by the extreme space environment. Since none of these factors are taken into account by the described standard testing conditions, including concentrations of required bacterial suspensions, a conclusive interpretation of the MIC is not possible in regard to resistance or susceptibility.

While sterile work environments have been designed for the cultivation of microorganisms on the ISS [74], there still remains the issue of the previously mentioned standard testing conditions.

While it may not be possible to quickly develop a clinical breakpoint for a spaceflight environment, it might be feasible to work towards an epidemiological cut-off (ECOFF) for the most important pathogens, such as the ESKAPE pathogens [75]: These pathogens comprise *Enterococcus faecium*, *S. aureus*, *Klebsiella pneumoniae*, *Acinetobacter baumannii*, *P. aeruginosa*, and *Enterobacter species*. All of them easily develop resistance against the first-line antibiotics used in their treatment. The ECOFF defines the normal distribution of MICs for each species and each tested antimicrobial substance without taking the clinical data into account. The ECOFF may not allow for susceptibility to be unambiguously defined, but at least it indicates antibiotic resistance [76,77].

Even after overcoming this issue, a standard testing environment must be defined and kept at hand throughout the flight. Again, this poses an immense challenge in terms of space availability, energy, time, expertise, and other resources. Yet, this could offer a solution for reproducible phenotypic resistance testing during spaceflight.

As already mentioned, there are several new procedures currently in (further) development [34], two of these prominent methods are genotypic resistance testing and MALDI-TOF. However, both still have their limits.

Genotypic resistance allows for quick results without incubation, and a Nanopore MinION DNA sequencer has been successfully tested onboard the ISS [78]. This indicates that molecular multiplex panels could be a solution for spaceflight. In addition to screening the most common resistance markers, they also allow for the identification of the causative pathogen. Furthermore, when cartridge-based, they are easy to handle [79] with a low risk of contamination. However, since detection is limited to the pathogens, and resistance determinants are included in the panel, they harbor the risk of misdiagnosis [80]. Previous studies showed an enhanced genotypic adaptation under spaceflight conditions, especially in genes related to stress response [5], as well as potentially higher mutation rates caused by radiation exposure [73]. This might also increase the risk of new mutations altering susceptibility that may not be covered in the panels. Furthermore, certain changes, for example, alterations in cell wall thickness as observed in *S. aureus* during the Soyuz 7 flight, could not be detected reliably [81]. Hence, genotypic testing is not always in line with phenotypic susceptibility testing. For example, wild-type strains may harbor resistance genes without them being expressed [82]. This leads to the predicament of how to interpret these contradicting results [83]. Furthermore, especially in *P. aeruginosa*, which has a whole depository of resistance mechanisms [84,85], fast changes in susceptibility patterns during therapy are likely to occur [86]. This means that certain time constraints need to be met, and it implies potential re-testing in short intervals. Both pose substantial challenges within the spaceflight context.

At the same time, assessment of antimicrobial resistance via mass spectrometry also seems to be a promising approach [87,88,89,90]. Simultaneously, as stated, miniaturization of the currently large devices is rapidly progressing. Small mass spectrometers have been successfully deployed in space exploration [61].

However, while both methods bear great potential, they are currently not routinely in use in clinical diagnostics.

## 4. Targeted Antimicrobial Therapy

Once again, there is the question of i.v. or p.o application. Assuming that the pathogen was identified as *P. aeruginosa*, there are several additional factors that can be highlighted by its therapy, which can also be applied to other antibiotic therapies against other species and during empiric therapy (Figure 1C).

As previously stated, *P. aeruginosa* is intrinsically resistant to several antibiotic classes, including, amongst others, aminopenicillins, cephalosporins up to class 3a, and trimethoprim-sulfamethoxazol. In terms of oral therapy, there is only one option: Fluoroquinolones, such as ciprofloxacin. However, this drug class has been critically evaluated more and more due to its side-effect profile. These include, but are not limited to, musculo-skeletal damage, such as an increased risk of tendon rupture, and affection of the central nervous system. Over the past few years, warnings have been issued by the regulatory institutions of several countries [91,92,93]. In the context of the spaceflight environment, this poses two important questions.

Firstly, what trade-off is acceptable in terms of easy oral administration that may include severe side effects versus an intravenous application of another substance? This substance might have a better side-effect profile, but the intravenous cannula poses an additional infection risk [94]. This might not only depend on general considerations, but also on the exact point within a mission. In other words, there can be no overall answer, but a meticulous evaluation needs to be made for each individual case. Once again, the incapacity of having readily available contact with experts on Earth will further impede decision-making.

Secondly, does the frequency of these side effects vary due to the already present physiological changes? This question intertwines with the following general thoughts on antibiotic therapy illustrated by the hypothetical *P. aeruginosa* UTI and its treatment.

When treating an infection, bioavailability and metabolization—that is, pharmacokinetics and pharmacodynamics—are of tremendous importance. The antibiotic administered needs to reach a certain level at the target site, i.e., the infection focus. Depending on the substance, this either refers to the peak concentration or the concentration over time [95]. This depends on the antimicrobial substance itself, but also on the causative pathogen and the type of infection. Individual factors, such as metabolic rate [96], further complicate matters, and include potential factors involving the initially mentioned oral prodrug pivmecillinam [97,98]. Apart from meropenem, an increased dosage of the primarily used antibiotics is usually required for the treatment of *P. aeruginosa* [70]. Depending on the resistance profile, severity of infection, and other influencing factors, combination therapy might further work towards therapy success [99,100].

As recently illuminated, compartment shifts and other physiological changes during reduced gravity conditions might greatly impact pharmacokinetics and dynamics [101,102]. Hence, an altered bioavailability needs to be taken into account, since the distribution volume may differ. This increases the risk of subtherapeutic drug levels and, thus, there is a risk of resistance development [103]; this also results in an increased risk of more pronounced side effects due to unnecessarily high concentrations.

In short, drug monitoring would be desirable, but once more, this requires immense amounts of expertise, training, and time. Drug monitoring itself should also be considered when thinking about potential antibiotics to be used during spaceflight. Glycopeptides [104], such as vancomycin, are notoriously difficult to adjust in concentration, especially in the first few days. Higher concentrations pose the risk of renal failure. This, again, may be further aggravated due to the already present compartment shifts and osmoregulation, including an altered renin–angiotensin–aldosterone system during spaceflight [105].

Ultimately, the above considerations perfectly intertwine with the previous thoughts on determining clinical breakpoints for antimicrobial substances. Understanding the changes in drug availability and compartmental distribution would contribute to determining the appropriate MICs.

In addition, the effect of the space environment on drug degradation and shelf life needs to be considered [106]. Altered environmental conditions, such as increased radiation exposure, may adversely affect drug stability, as is the case with trimethoprim-sulfamethoxazole [107].

## 5. Re-Testing and Therapeutic Success

The final stage of the case tackles the questions of potential re-testing and duration of antimicrobial therapy. Especially with the assumed pathogen *P. aeruginosa*, resistance is prone to develop rapidly during therapy. This is due to the underlying mechanisms, such as changes in the outer cell membrane [108]. Hence, time points during therapy at which a repetition of the susceptibility testing seems appropriate due to the risk of therapeutic failure need to be defined. To do so, we need to understand how the space environment might affect the cellular processes involved. As this differs between species—not the least because of differing underlying resistance mechanisms—initially concentrating on the already mentioned ESKAPE pathogens [75] seems to be a workable and realistic approach (Figure 1D).

In regard to the antimicrobial treatment itself, therapy duration is a current topic of debate, with recent publications indicating no adverse effects for shorter durations and early oral administration for several infections [109,110]. This also has to be considered in the spaceflight environment, bearing the additionally discussed factors in mind.

In our case, after going through each of the obstacles discussed, we can conclude that everything ended well. The astronaut was treated successfully, and the mission proceeded as planned.

## 6. Outlook and Conclusions

This essay presents a thought experiment that has been of a rather practical and fundamental nature. However, it may help to discover pitfalls and elucidate how even an easily treatable infection on Earth could have rather drastic consequences in space when considering rational antibiotic therapy in astronauts.

In addition, this approach could be applied to other infections, such as respiratory and central nervous infections. Following this notion, there are some problems common to all these conditions. However, for some, transmission or sampling may differ substantially, posing new challenges and obstacles.

This becomes evident when looking at other pathogen groups.

When looking at viral infections, such as COVID-19 and influenza, PCR-based diagnostic and antigen tests allow for relatively easy implementation as POCT [111]. Therapy, depending on the severity of an illness, may include, amongst others, antiviral substances, glucocorticoids, and antibodies [112]. This, again, poses unique challenges in the provision of these substances during spaceflight, as well as in the decision of when to apply them. In addition, while the previously mentioned pre-flight vaccinations help to prevent infection, more data is required on potential booster schemes during spaceflight.

Diagnosing fungal infections, such as invasive candidiasis, is often difficult, requiring a combination of clinical examination, culture-based methods, potential PCR-based methods, and, depending on suspected organ manifestations, adequate imaging procedures. Biomarkers, such as ß-D-Glucan, may further assist diagnosis [113].

These aspects are even more pronounced when looking at polymicrobial infections. Apart from potentially requiring an array of diagnostic tools, substantial expertise in infectious disease is required to an even greater extent to ensure adequate medical care throughout the whole infection process. For example, superinfection with *S. aureus* in Influenza A patients is related to more severe cases. Similarly, *Candida (C.) albicans* and *S. aureus* can aggravate the infection due to their interactions, such as *C. albicans* facilitating the entry of *S. aureus* by damaging the organ barriers. Hence, microbe–microbe interactions, whether they be synergistic or competitive, have to be taken into account when assessing the progression of the infection [114].

To summarize, the following issues have become most apparent during the steps taken above. Apart from an apparent lack of data on large numbers of strains and their antibiotic resistance in space environments, there is also the amount of knowledge and expertise needed from astronauts to effectively diagnose and treat infections that should be considered. Hence, including crew members with professional and broad medical backgrounds would be desirable.

Next, the available space within the spacecraft, obtaining standard testing conditions, and the shelf life of the required substances, including antibiotics [115], must be considered. [115] Production of antibiotics and other substances seems inevitable. To achieve this, great potential lies in using microbiology itself, for example, by using strains to biomanufacture desired compounds [116]. Furthermore, potential also lies in packaging, alternative application technologies, and the development of drugs that exhibit higher resistance to radiation exposure [117].

Lastly, the constraints of our current technologies remain. As already stated, there are fast-progressing developments, but currently, bacterial cultures remain state-of-the-art for many common pathogens.

**Figure 2:**
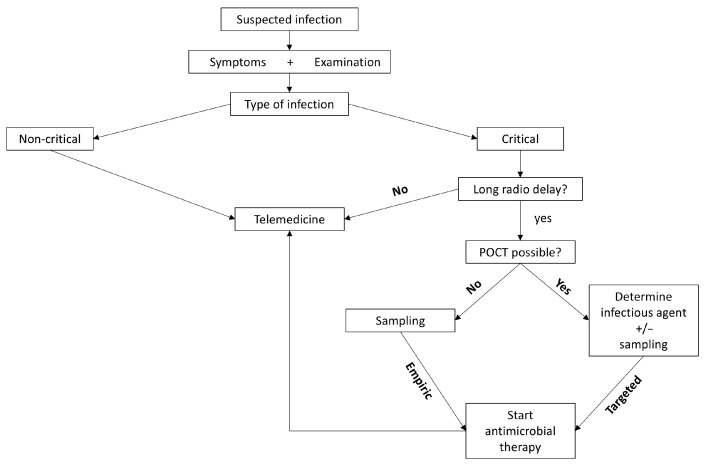
Decision tree depicting the main aspects of infection diagnostics. After initial evaluation by the crew medical officer, further proceedings depend on the type of infection and potential radio delay [POCT: point-of-care testing].

However, there are solutions to all these queries that can be, or are already being, addressed today (Figure 1E):

In terms of diagnosing and treating infections in space, individualized medicine is most definitely a part of the solution [118,119]. It might also help to overcome the previously explained obstacle of drug monitoring [120]. New diagnostic tools, such as biomarkers [121], in combination with the already discussed POCT, might augment diagnostic and therapeutic management. However, the current core of infection management remains the crew medical officer in combination with telemedicine and its potential to access Earth-based medical experts (Figure 2). Yet, as the distance from Earth increases, human expertise can only be assessed with an immense time delay. Artificial intelligence [122] and machine learning [123,124] may assist astronauts in dealing with critical infections, resulting in more autonomous diagnostic tools [125]. Moreover, they may help to decide whether an infection is highly critical in the first place.

Next, there is a need to select antibiotics that are vital during long-term spaceflight. The decision should be based on their antimicrobial spectrum, application, drug monitoring, side effects, propensity to resistance development, and shelf life in space. An important point, which is difficult to foresee, is the severity of the infections that need to be treated. This aspect has, so far, only been mentioned marginally. One example, to illustrate the importance, is a bloodstream infection with methicillin-susceptible *Staphylococcus* (*S*.) *aureus*. The treatment of choice would be cefazolin or flucloxacillin. However, side effects, including nephrotoxicity, are more pronounced for the latter [126,127]. Both substances cover only a small spectrum of bacterial infections; however, *S*. *aureus* bloodstream infections account for high morbidity and mortality rates [128]. Since *S. aureus* is also a potential commensal of the skin and other locations [129,130], there may remain an infection risk, despite potential decolonization measures. In addition, *S. lugdunensis*, a coagulase-negative *staphylococcus* that is also a skin commensal, has the potential to cause similar severe infections as *S. aureus* [131]. Hence, cefazolin seems to be an obvious choice to consider for long-term spaceflights.

Apart from the classical antimicrobial therapies, new strategies are quickly evolving [132], such as phage therapy [133,134]. Moreover, there remains the large field of infection prevention, including various approaches such as probiotics [135], vaccination developments [136], and antibiotic stewardship [137]. After all, the best way to manage infections is to prevent them. This also concurs with the pre-flight protocols briefly mentioned in the introduction.

Yet, given the apparent connection between physiological changes and our limited understanding of infections under spaceflight conditions thus far, whenever possible, the already applied microbiological sampling and monitoring [9] should be continued to be included in future studies, whether it be directly in spaceflight or in Earth-based simulation. For the latter, long-term isolation studies simulating long-term flights, such as Mars-500 and the Green Star 180 project, not only help to gain important data about the effect of confined spaces on the microbiota [138,139], but also allow simulation of the issues discussed above. This, ultimately, can contribute to the development of the best practice procedure for infections during spaceflight.

As presented, elucidating this interconnectivity will inevitably be an important component for safe long-term spaceflight. In addition, screenings of large strain numbers of various species under real or simulated spaceflight conditions are vital to assess the antimicrobial resistance of the wild-type bacterial population. The already mentioned limitation to classical pathogens will most likely result in the highest benefit over the shortest period of time.

The question of what risks are deemed to be within reasonable boundaries also remains. For example, the above-described scenario does not account for fastidious growing organisms. These organisms are often difficult to assess, even under standard laboratory conditions.

In conclusion, the advances in medical microbiology and infectious diseases needed to guarantee successful advancement in human spaceflight may also offer the chance to advance Earth-based operations [140] and, ultimately, patient care.

## Abbreviations

The following abbreviations are used in this manuscript:

C.	Candida
ECOFF	Epidemiological cut-off
MIC	Minimum inhibitory concentration
ISS	International Space Station
i.v.	Intravenous
P.	*Pseudomonas*
p.o.	Per os
POCT	Point-of-care testing
S.	*Staphylococcus*
UTI	Urinary tract infection
